# Intercostal misplacement of a thoracic epidural catheter discovered during lung cancer surgery: a case report

**DOI:** 10.1186/s40981-019-0264-8

**Published:** 2019-07-11

**Authors:** Izumi Kawagoe, Masakazu Hayashida, Daizoh Satoh, Eiichi Inada

**Affiliations:** Department of Anesthesiology and Pain Medicine, Juntendo University School of Medicine, 2-1-1 Hongo, Bunkyo-ku, Tokyo, 113-8421 Japan

**Keywords:** Epidural catheter misplacement, Thoracic epidural analgesia, Lung surgery, Intercostal space

## Abstract

**Background:**

Blind epidural catheter placement can lead to inadvertent misplacement. We present a case of intercostal misplacement of a thoracic epidural catheter.

**Case presentation:**

A 67-year-old male underwent left lung cancer surgery via thoracotomy with epidural analgesia via the Th 5–6 intervertebral space, although with some difficulty. We detected dermatomal cold sensory loss around Th five min after initial administration of local anesthetics through the catheter before general anesthesia induction. However, the epidural catheter was intraoperatively found below the fifth rib, running along the course of the intercostal nerve. The catheter was successfully withdrawn via his back, and we postoperatively performed paravertebral block under ultrasound guidance. He did not complain of complications at discharge.

**Conclusions:**

Detailed bilateral assessment of sensory loss after initial local anesthetic administration might have facilitated preoperative detection of the misplacement. In cases requiring multiple catheter insertion attempts, switching to another analgesic method should be considered.

## Background

Thoracic epidural anesthesia (TEA) is the gold standard postoperative analgesic method for thoracic surgery [1]. Although thoracic paravertebral block (TPVB) and erector spinae plane block (ESPB) under ultrasound guidance are popular postoperative analgesia methods, epidural catheter placement by a blind technique is still the most common procedure practiced by anesthesiologists for postoperative analgesia after thoracic surgery. We present a case of intercostal misplacement of a thoracic epidural catheter, which was identified visibly in the surgical field.

## Case presentation

The patient gave written informed consent for publication of this case report and any accompanying images, and the presentation was approved by the institutional review board of Juntendo University (JHS 18-027).

A 67-year-old male, 170 cm tall, weighing 72 kg, was scheduled to undergo left lower lung lobectomy via open thoracotomy for non-small cell lung carcinoma. He had no relevant medical history, and his preoperative blood examination revealed no abnormal findings. We decided to preoperatively insert a thoracic epidural catheter for postoperative analgesia.

On the day of surgery, no premedication was administered. He was monitored by ECG, noninvasive blood pressure monitoring and pulse oximetry. Before general anesthesia induction, the patient was positioned in the right lateral position for catheter insertion. We initially attempted the epidural puncture at the thoracic (Th) 6–7 interspace, which we changed to the Th 5–6 interspace after 15 min due to difficulty in an epidural puncture at the original site. Using a right paramedian approach, an 18 G Tuohy needle (Perican® epidural needle, 18G × 80 mm, B. Braun, Hessen, Germany) was used for epidural puncture 1 cm caudal and 1 cm to the right of the spinous process. After confirming accurate placement of the needle tip in the epidural space by the loss of resistance to injection of normal saline, the epidural catheter (Perifix® epidural catheter, standard 1000 mm, B. Braun) was inserted via the Th 5–6 intervertebral space with no resistance. The final total insertion length was 13 cm: 6 cm through the skin and 7 cm placed in the epidural space. After the catheter was appropriately secured in place, the patient was turned to the supine position in preparation for general anesthesia. Before general anesthesia induction, we gave a test dose of 2 ml of 2% plain lidocaine via the epidural catheter, followed by administration of 50 μg fentanyl and 4 ml of 0.25% levobupivacaine. We were able to confirm the loss of cold sensation around the left T5 area 5 min after local anesthetic administration. After induction of general anesthesia, endotracheal intubation with a double-lumen tube was performed to enable single lung ventilation during surgery.

**Fig. 1:**
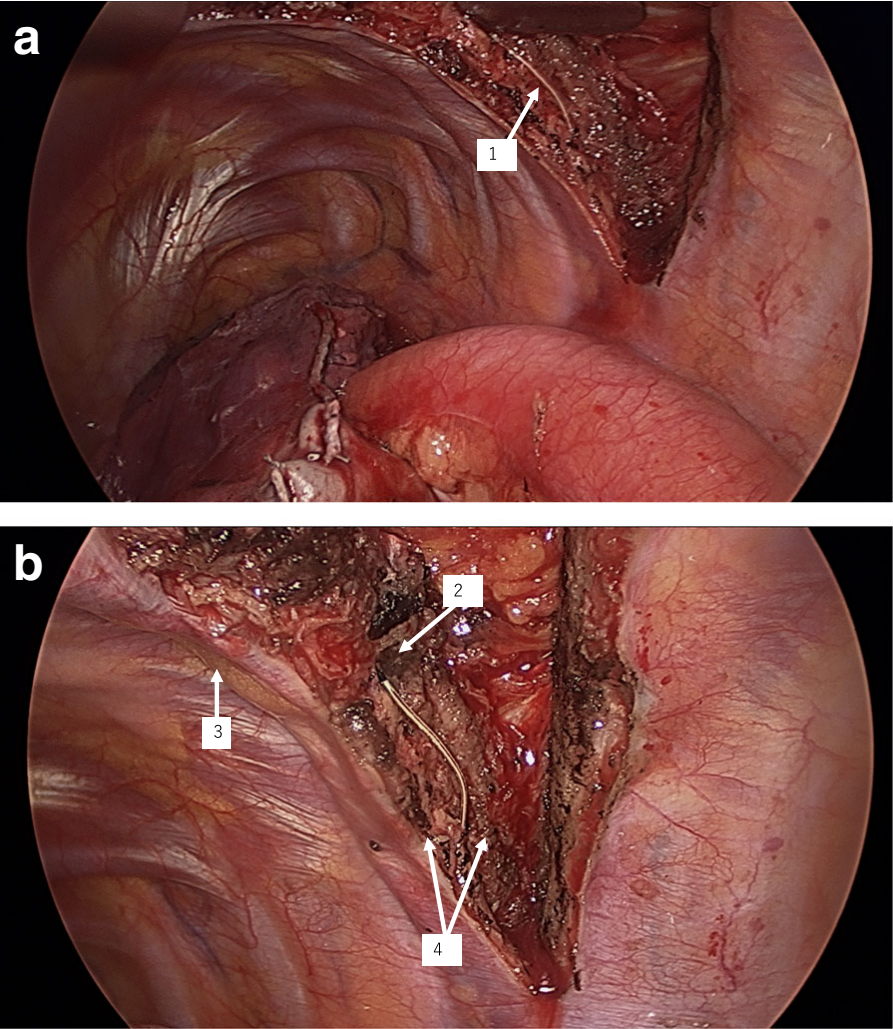
**a**, **b** The epidural catheter is seen 3 cm from the dorsal edge of the posterolateral incision at the Th 5–6 intercostal space. Arrow 1, the epidural catheter was found between the innermost and internal intercostal muscles, below the fifth rib. It seemed to run along with the intercostal nerve. Arrow 2, tip of the catheter. Arrow 3, fifth rib. Arrow 4, the innermost and internal intercostal muscles

At the beginning of surgery, an approximately 12- cm posterolateral incision was made at the level of the fifth to sixth intercostal space. Blood pressure and heart rate remained stable, suggesting the adequacy of analgesia in the incision area. However, when the surgeon opened the thoracic cavity, he saw the epidural catheter below the fifth rib. The catheter seemed to follow the course of the intercostal nerve. The tip of the catheter could be seen 3 cm from the dorsal edge of the incision (Fig. 1a, b).

Fortunately, the catheter had not been severed during the surgical procedure. After discussion with the surgical team, we decided to withdraw the catheter via the skin of his back under direct observation by the surgeon in the surgical field. The catheter was successfully withdrawn and examined for intactness of the tip. Thereafter, intraoperative analgesia was achieved mainly by continuous intravenous infusion of remifentanil and intermittent administration of fentanyl. For postoperative analgesia, a continuous intravenous infusion of 1 mg (20 ml) fentanyl and 20 ml normal saline was administered as patient-controlled analgesia (PCA) at the rate of 1 ml/h, with bolus doses of 1 ml and a lockout time of 10 min, the infusion being commenced in the latter half of surgery. The scheduled surgery was completed uneventfully without any complications.

We also performed TPVB under ultrasound guidance, injecting 20 ml of 0.25% levobupivacaine in the paravertebral space, besides the Th5 transverse process, before the patient’s emergence from general anesthesia and while he was still in the lateral position.

Anesthesia emergence and his subsequent recovery from anesthesia were uneventful. During the postoperative period, his numerical rating scale score ranged from 0 to 1 and he required a single administration of supplemental analgesic (15 mg pentazocine) and six IV PCA boluses within 24 h. He did not complain of any paresthesia around the intercostal region. He was discharged from the hospital without any paresthesia or complications on the fifth day after surgery.

## Discussion

Thoracotomy patients, in particular, as compared to patients who undergo video-assisted thoracic surgery or robot-assisted thoracic surgery, require TEA due to the severe acute pain associated with open thoracotomy [2]. Moreover, TEA reduces postoperative pulmonary complications [3]. Recently, TPVB and ESPB have also become available for post-thoracic surgery analgesia. These blocks are performed under ultrasound guidance in most cases. The technique of epidural catheter insertion under ultrasound guidance is reported to be more accurate than the blind technique in pediatric patients [4]. However, TEA under ultrasound guidance is not common in adult patients.

Catheter misplacement, although rare, is a known complication of epidural catheter insertion. In most such cases, the catheter is advanced into the intrathoracic cavity or intrapleural space and is identified during thoracic surgery [5]. Although confirmation of misplacement of a TPVB catheter into the epidural space by postoperative radiographic examination has been reported [6], our case of misplacement of an epidural catheter into the paravertebral space represents a central to peripheral misplacement. Referring to the insertion length and the length of catheter that was found in the surgical field, it is likely that the catheter was inserted through the paravertebral space and not the epidural space. Lumbar epidural catheter misplacement is seldom recognized during surgery. Rajira et al. [7] reported a case of right-sided open nephrectomy, in which the catheter was found in the surgical field emerging from the psoas major muscle.

In previously reported cases of intercostal misplacement of thoracic epidural catheters, the catheter was rolled up [8] or ran straight [9] in the intercostal space, although it was discovered after incision as in our case. However, the previous reports did not mention any analgesic effect of the catheter. Since we inserted the catheter to a depth of 7 cm in the epidural space and about 3 cm of the catheter was visible along the direction of the intercostal incision, the remaining 4 cm of the catheter was probably located posterior to the incision. Consideration of the distance between the incision edge and vertebral midline, which was probably more than 4 cm, suggests that the catheter might have been inserted into the paravertebral space. This mis-insertion could have been because we selected the paramedian approach from the right side for epidural puncture. After the catheter was inserted via the epidural or paravertebral space, it was thought to run in a peripheral direction between the innermost and internal intercostal muscles, closely following the course of the posterior branch of the intercostal nerve below the fifth rib [10]. The stable vital signs at the time of skin incision in our patient could have been due to inadvertent intercostal nerve block by the drugs injected via the epidural catheter, which prevented us from noticing the misplacement until the surgeon’s comment. Fortunately, the entire catheter could be pulled out without it being severed or causing any damage. In our case, although we observed that the catheter had been advanced in the direction of the intercostal nerve, the patient did not complain of any paresthesia or abnormal feeling during the epidural procedure, which had been performed while he was completely conscious.

The epidural puncture took a long time in our case because of the presence of anatomical deformity of the spine and lack of flexibility, rather than any technical problem. We should ideally have switched to another analgesic method, such as TPVB or ESPB under ultrasound guidance, after several attempts at catheter insertion. Attempting epidural catheter insertion after induction of general anesthesia would have been another option, since the resultant muscle relaxation might have increased his flexibility, facilitating catheter insertion. However, we do not normally insert epidural catheters under general anesthesia in adult patients, because it is impossible for unconscious patients to notice any paresthesia during catheter insertion by the blind technique. Moreover, if we had taken adequate time after initial administration of local anesthetics through the catheter to check for a wider area of sensation loss, we would probably have noticed the misplacement in the intercostal space before general anesthesia induction.

This case proves that intercostal misplacement of epidural catheters inserted by the blind technique actually occurs, although extremely rarely. The catheter was, fortunately, successfully withdrawn without any complications. Detailed dermatomal testing of sensory loss after initial drug administration through the catheter might have helped detect misplacement. Our experience also suggests that it might be prudent to switch to another analgesic method after several failed attempts at epidural catheter insertion.

## Data Availability

Not applicable.

## Abbreviations

ESPB: Erector spinae plane block
IV-PCA: Intravenous patient-controlled analgesia
TEA: Thoracic epidural analgesia
TPVB: Thoracic paravertebral block
